# 5mC and 5hmC dynamics during PGC reprogramming and role of Tet1 in female meiosis

**DOI:** 10.1186/1756-8935-6-S1-P89

**Published:** 2013-03-18

**Authors:** Shinpei Yamaguchi, Kwonho Hong, Rui Liu, Li Shen, Azusa Inoue, Dinh Diep, Kun Zhang, Yi Zhang

**Affiliations:** 1 Harvard Medical School and Boston Childrens Hospital, Boston, USA

## 

Meiosis is a germ-cell-specific cell division process through which haploid gametes are produced for sexual reproduction. Before the initiation of meiosis, mouse primordial germ cells undergo a series of epigenetic reprogramming steps, including the global erasure of DNA methylation at the 5-position of cytosine (5mC) in CpG-rich DNA. However, the 5mC dynamics and its relationship with the generation of 5-hydroxymethylcytosine (5hmC) during the reprogramming process are not clear. Here we analyzed the dynamics of 5mC and 5hmC during PGC reprograming. Unexpectedly, we found a “blank period” (E8.5-9.5) in which both 5mC and 5hmC are low. After that, 5hmC level increases and reaches its peak at E10.5 and gradually decreases until E13.5. Interestingly, 5hmC is enriched in chromocenter during this period in a Tet1 -dependent manner. While this germ cell-specific 5hmC subnuclear localization pattern is maintained in female germ cells till mature oocytes, such pattern is gradually lost in male germ cells as mitosis resumes at neonatal stage. Using a loss-of-function approach in mice, we show that Tet1 has an important role in regulating meiosis in mouse oocytes. Tet1 deficiency significantly reduces female germ-cell numbers and fertility. Univalent chromosomes and unresolved DNA double-strand breaks are also observed in Tet1-deficient oocytes. Tet1 deficiency does not greatly affect the genome-wide demethylation, but leads to defective DNA demethylation and decreased expression of a subset of meiotic genes. Our study thus not only reveals the dynamics of 5mC and 5hmC during PGC reprogramming, but also demonstrate a function for Tet1 in meiosis and meiotic gene activation in female germ cells.

